# Characterization of infiltrating lymphocytes in human benign and malignant prostate tissue

**DOI:** 10.18632/oncotarget.19528

**Published:** 2017-07-24

**Authors:** Emelie Rådestad, Lars Egevad, Carl Jorns, Jonas Mattsson, Berit Sundberg, Silvia Nava, Bo-Göran Ericzon, Lars Henningsohn, Victor Levitsky, Michael Uhlin

**Affiliations:** ^1^ Department of Clinical Science, Intervention and Technology, Karolinska Institutet, Stockholm, Sweden; ^2^ Department of Oncology-Pathology, Karolinska Institutet, Stockholm, Sweden; ^3^ Department of Pathology, Karolinska University Hospital, Stockholm, Sweden; ^4^ Centre for Allogeneic Stem Cell Transplantation, Karolinska University Hospital, Stockholm, Sweden; ^5^ Department of Urology, Karolinska University Hospital, Stockholm, Sweden; ^6^ Molecular Partners AG, Schlieren-Zurich, Switzerland; ^7^ Department of Immunology/Transfusion Medicine, Karolinska University Hospital, Stockholm, Sweden; ^8^ Department of Applied Physics, Royal Institute of Technology, Stockholm, Sweden

**Keywords:** prostate cancer, benign prostatic hyperplasia, prostate-infiltrating lymphocytes, checkpoint blockade, PD-1

## Abstract

Immune checkpoint blockade has shown promising results in numerous cancer types. However, in prostate cancer (PC), absent or limited responses have been reported. To investigate further, we compared the phenotype of infiltrating T-cells isolated from prostate tissue from patients with PC (*n* = 5), benign prostatic hyperplasia (BPH) (*n* = 27), BPH with concurrent PC (*n* = 4) and controls (*n* = 7). The majority of T-cells were CD8^+^ and had a CCR7^−^CD45RO^+^ effector memory phenotype. However, the yield of T-cells isolated from PC lesions was on average 20-fold higher than that obtained from control prostates. Furthermore, there were differences between the prostate conditions regarding the percentage of T-cells expressing several activation markers and co-inhibitory receptors. In conclusion, many prostate-infiltrating T-cells express co-inhibitory receptors PD-1 and LAG-3, regardless of prostate condition. Despite the observed increase in counts and percentages of PD-1^+^ T-cells in PC, the concomitant demonstration of high percentage of PD-1^+^ T-cells in control prostates suggests that PD-1 may play a role in controlling the homeostasis of the prostate rather than in contributing to PC-associated immune-suppression. Thus, PD-1 may not be a good candidate for checkpoint blockade in PC and these data are relevant for evaluation of clinical trials and in designing future immunotherapeutic approaches of PC.

## INTRODUCTION

The healthy human prostate contains various types of immune cells, however knowledge is limited regarding their function within the prostate environment [1, 2]. Chronic inflammation is common in the prostate and is increasingly discussed as a driver of both benign and malignant conditions [3]. The causes of chronic prostatic inflammation are debated and still not well understood [1, 4], however the increased inflammation-induced immunological presence is complex and can have dual roles in the outcome. Primarily, the immune cells are required for surveillance, neutralization and clearance of foreign pathogens and transformed cells within the prostate environment. However, their phenotype and function can become altered by the inflammatory environment itself, affecting their primary purpose. With increased cellular damage, release of growth factors, etc., the inflammatory environment can promote development of prostate pathology. The link between inflammation and cancer is widely accepted in numerous cancer types, including prostate cancer (PC) [3–7], and is today described as an enabling characteristic of tumor progression [8–11].

PC is currently the second most common cancer type among men worldwide [12]. Despite earlier detection and improved treatments, it remains the second leading cause of cancer-related deaths among men [12]. Other non-malignant conditions of the prostate, such as benign prostatic hyperplasia (BPH), have high prevalence in older males [13]. In BPH, T-cells have been suggested to polarize towards Th2 and Th17, at the cost of decreased tumor-directed Th1 responses [1, 14]. To understand the implications of immune infiltration in PC, it is crucial to characterize this infiltration in both PC and non-malignant prostate conditions.

It is important to elucidate how the immune system is involved in the development of pathologic conditions of the prostate to enable effective evaluation of emerging immunotherapeutic approaches, such as checkpoint blockade. This approach aims to restore and enhance the commonly suppressed anti-tumor capacity of tumor-infiltrating lymphocytes (TILs). Current clinical trials of checkpoint blockers, including anti-CTLA-4 and/or anti-PD1 have shown promising results in many different cancer types. Their success in PC patients have so far been absent or limited in comparison to other cancer types such as metastatic melanoma and non-small cell lung cancer [15, 16]. However, a recently published phase II study including ten metastatic castration-resistant PC patients re-opened the possibility of using PD-1 blockade in PC [17]. In this study, objective clinical response activity was shown for the first time using an anti-PD-1 targeting agent. The factors driving the success rate of checkpoint blockade remain largely unknown and prediction of responses in different cancer types, patient groups and selection of the best combination treatments requires additional evidence-based guidelines.

Using immunohistochemistry, it has been shown that PC lesions are surrounded by lymphocyte clusters expressing the co-inhibitory receptor PD-1 and its ligand PD-L1, as well as markers associated with regulatory T-cells (Tregs) [18]. Sfanos *et al*. have showed that enzymatically isolated CD8^+^ T-cells from prostate tissue cores of PC patients were primarily PD-1^+^ [19]. Both these studies speculate that that functional inhibition and exhaustion negatively affects the effector functions of prostate-infiltrating lymphocytes. Several other studies have reported the presence of hematopoietic cells in PC lesions but have been unable to provide a detailed subset phenotyping and functional characterization [4]. In fact, there are few published studies on freshly isolated human prostate-infiltrating immune cells, likely due to challenges in tissue collection and processing. Here, we set out to investigate the phenotype of TILs in PC by characterizing freshly isolated immune cells with multicolor flow cytometry.

To enable identification of tumor-specific phenotypes, we sought to compare the composition of lymphocytes at two different locations, one malignant and one non-malignant site, of the same prostate removed by radical prostatectomy. Furthermore, we wanted to compare the phenotype of immune cells in PC with our previously published findings in BPH tissue [20]. Lastly, we wanted to compare our findings with adequate control material from deceased organ donors. In the latter material, the aim was to collect infiltrating immune cells from the entire prostate as representation of the steady-state immune status of the organ.

The overall goal of the current study was to increase the knowledge about the phenotypes of immune cells present in different prostate conditions, including PC, with the focus on T-cells and their co-inhibitory receptor expression. Our results may help to further develop and refine approaches toward immunotherapy of PC.

## RESULTS

### Quantification of three major lymphocyte populations in prostate tissue

**Figure 1:**
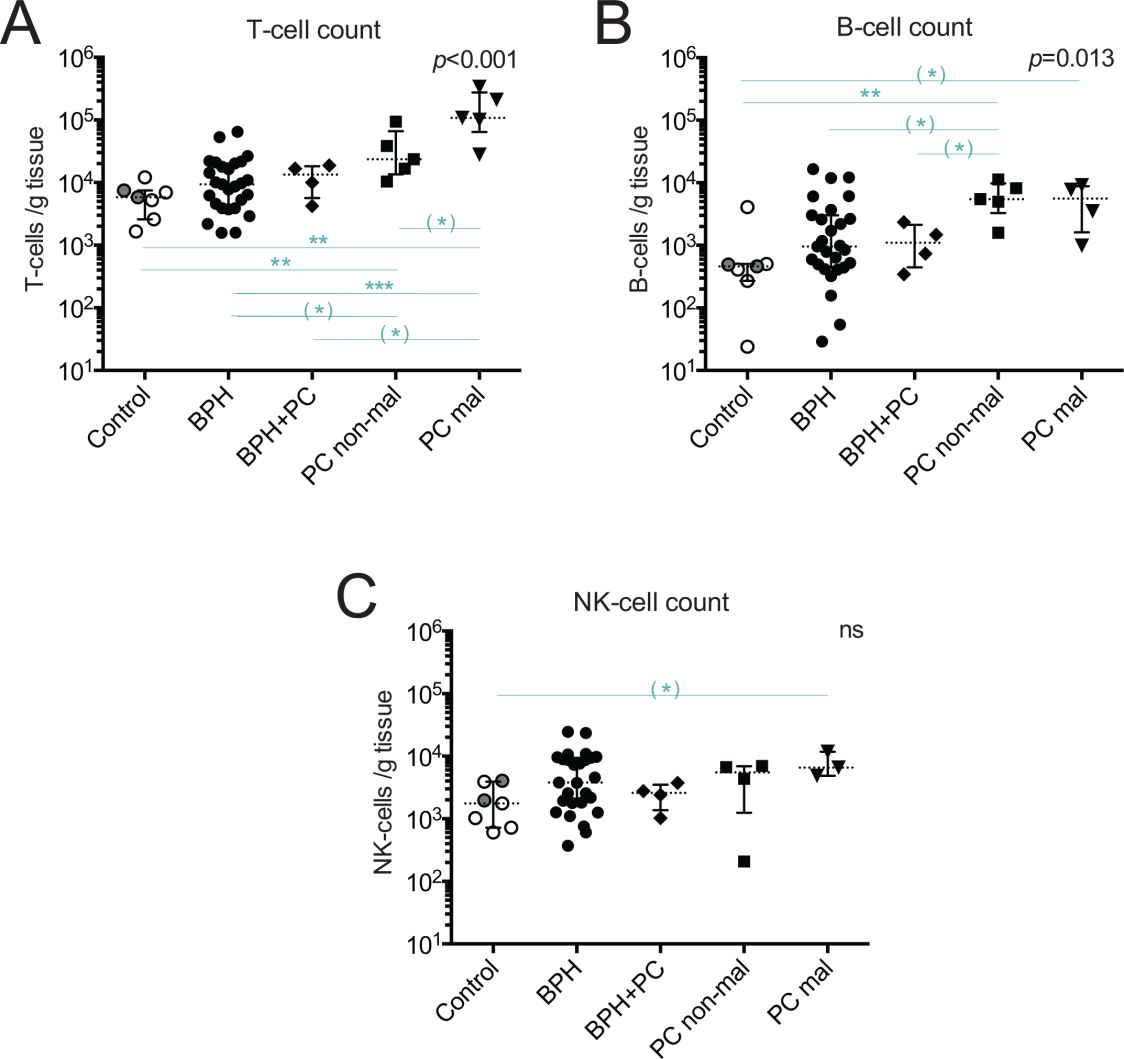
Quantification of major lymphocyte subsets isolated from five prostate conditions Number of (**A**) T-cells (CD3^+^ events); (**B**) B-cells (CD3^−^/CD19^+^ events); and (**C**) NK-cells (CD3^−^/CD56^+^ events) in different prostate conditions per gram processed tissue. Median and interquartile ranges are shown for all. Presented *p*-values are from Kruskal Wallis tests and the horizontal lines present results of post-hoc Mann-Whitney *U*-tests. With Bonferroni correction and significance level of *p <* 0.01, the results initially showing *p <* 0.05 are considered as trends and are marked with (*). **p < 0.01; ***p < 0.001, ns = no significance. Among the control prostates, the results of the two youngest donors are marked in dark grey circles.

The composition of prostate-infiltrating lymphocytes freshly isolated from tissue samples representative for the following five different prostate conditions was analyzed using multicolor flow cytometry: control tissue from deceased organ donors, BPH, BPH with concurrent PC (hereafter referred to as BPH+PC), and two sites from prostates of PC patients; one malignant and one non-malignant site. Quantification of T-, B- and NK-cells was done for each sample and the integrated results compared for the indicated sample types (Figure 1).

The median yield of T-cells in malignant PC samples was nearly 20 times higher compared to control, 11 times higher compared to BPH, 8 times higher compared to BPH+PC, and 5 times higher compared to non-malignant PC samples (Figure 1A). This increase in malignant PC samples was, after Bonferroni correction, significant compared to controls (*p* = 0.003, median 107 900 vs. 5 840 T-cells/gram) and BPH (*p* < 0.001, median 107 900 vs. 9 400 T-cells/gram). The other findings can be considered as strong trends (Figure 1A, significance level with Bonferroni correction was *p* < 0.01). The T-cell yield obtained in non-malignant PC samples was also significantly increased compared to normal controls (*p* = 0.005), approximately 4 times higher. Overall, the high degree of T-cell infiltration in malignant and non-malignant prostate tissue made it possible to isolate T-cells from a limited amount of material (median 0.03 grams from malignant site, Table 1).

**Table 1:** Prostate cancer patient characteristics and sample collection information

Patient #	Clinical factors		Pathology				Sample collection		
	Age	Pre s-PSA (μg/L)	Pathologic stage	Gleason score	Surgical margin	Extra prostatic extension	Non-malignant tissue (g)	Malignant tissue (g)	Peripheral blood (mL)
1	64	12	2	3 + 4 = 7	Negative	No	0.15	0.03	18
2	64	12	2	4 + 5 = 9	Positive	No	0.21	0.02	18
3	69	33	3a	3 + 4 = 7	Positive	Yes	0.03	0.03	18
4	64	13	2	3 + 4 = 7	Negative	No	0.09	0.05	18
5	71	10	3a	4 + 3 = 7	Negative	Yes	0.36	0.01	18

Abbreviations: pre s-PSA, serum prostate-specific antigen levels before time of surgery.

All cancers were of peripheral zone origin, clinically staged as T2 and none showed signs of seminal vesicle invasion.

Prostate infiltration of B- and NK-cells was much more limited compared to T-cells (Figure 1). B-cells were the rarest of the three evaluated lymphocyte subsets with a median of 5 610 B-cells/gram in malignant tissue and 460 B-cells/gram in control tissue, a 12-fold difference in yield between these two sample types (Figure 1B). Due to limited number of samples, only a strong statistical trend was observed between these two conditions (*p* = 0.024) but there was a significant increase in B-cell number in non-malignant tissue compared to controls (*p* = 0.005). The number of B-cells in control, BPH and BPH+PC appeared to be similar but there was a trend towards increased number of B-cells in non-malignant PC compared to both BPH (*p* = 0.035) and BPH+PC (*p* = 0.032) (Figure 1B).

Compared to B-cells, NK-cells were more abundant with a median of 6 570 NK-cells/gram in malignant tissue and 1 760 NK-cells/gram in control tissue (Figure 1C). NK-cell counts were in general more similar between the conditions but a strong trend towards increased NK-cell numbers in malignant tissue compared to control tissue was found (*p* = 0.017).

### General characterization of prostate-infiltrating T-cells

Next, we characterized and analyzed the frequencies of different T-cell subsets present in prostate-derived immune infiltrates. Expression was assessed as percentage gated from total CD3^+^ T-cells or on T-cell subsets (subgated on CD4^+^ or CD8^+^ T-cells).

**Figure 2:**
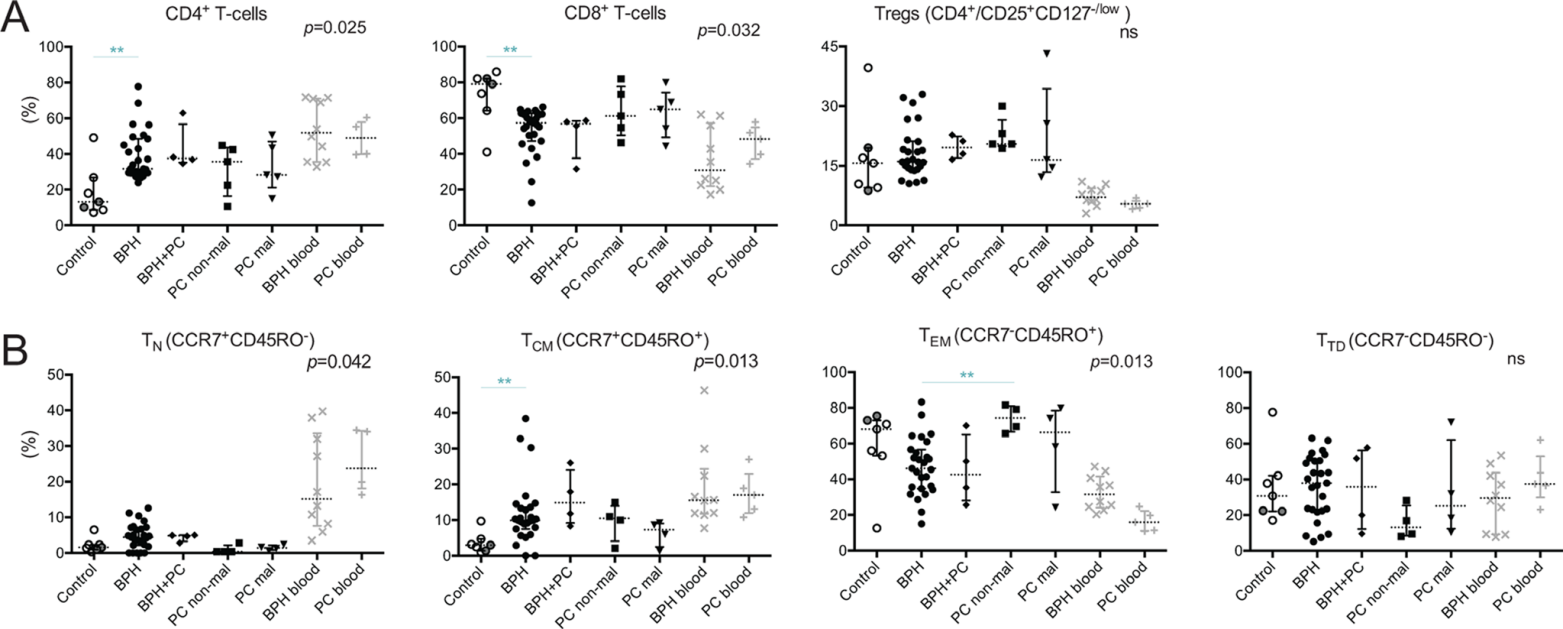
Percentages of general immune cell subsets in five different prostate conditions and peripheral blood (**A**) The percentages of CD4^+^, CD8^+^, and regulatory T-cells (Tregs) are presented. (**B**) Proportion of T-cells of four memory/maturation stages: T_N_ (naïve); T_CM_ (central memory); T_EM_ (effector memory); and T_TD_ (terminally differentiated) are shown. Median and interquartile ranges are shown for all. *P*-values for Kruskal-Wallis test on five prostate conditions are presented, peripheral blood values (BPH/PC blood) were excluded from all statistical analysis. Horizontal lines present the significant results of post-hoc Mann-Whitney *U*-tests. Due to multiple comparisons, Bonferroni correction was calculated and significance level of *p* < 0.01 was used. Values resulting in *(*p <* 0.05) were excluded, not plotted and considered as trends. **p < 0.01; ***p < 0.001, ns = no significance. Among the control prostates, the results of the two youngest donors are marked in dark grey circles.

The majority of prostate-infiltrating T-cells were CD8^+^ in all five prostate conditions (Figure 2A). The proportion of CD8^+^ T-cells was comparable in all conditions, except in the controls which showed a significantly higher presence of CD8^+^ T-cells compared to BPH (median 79.1% vs. 57.4%, *p* = 0.002). The majority of infiltrating T-cells had an effector memory phenotype, defined as CCR7^−^CD45RO^+^ (Figure 2B). The proportion of naïve T-cells (CCR7^+^CD45RO^−^) between the prostate tissue types was similar, all having a limited percentage compared to peripheral blood (Figure 2B). Central memory T-cells (CCR7^+^CD45RO^+^) were increased in BPH compared to control prostates and showed a large spread between different patients. Effector memory T-cells were significantly increased in the non-malignant PC lesions compared to BPH (*p* = 0.002). This pattern was similar for the malignant PC lesions but due to a larger spread, no significant difference was found. There was also a large spread in the percentage of terminally differentiated T-cells (CCR7^−^CD45RO^−^), similar to what was observed in peripheral blood, and there were no significant differences between the prostate conditions (Figure 2B).

The percentage of Tregs, defined as CD4^+^ T-cells expressing CD25^high^CD127^−/low^, was examined between the prostate conditions. Compared to peripheral blood, the percentage of Tregs was significantly elevated in all prostate tissue types; median 15.7%, 16.1%, 19.6%, 20.5% and 16.5% in control, BPH, BPH+PC, non-malignant and malignant site respectively compared to 7.2% and 5.4% in the blood of BPH and PC patients (Figure 2A). No significant differences or trends were observed between the different prostate conditions. There was a pronounced spread in proportion of Tregs at the malignant site of PC patients, ranging from 12.2% to 43.2% (Figure 2A).

**Figure 3:**
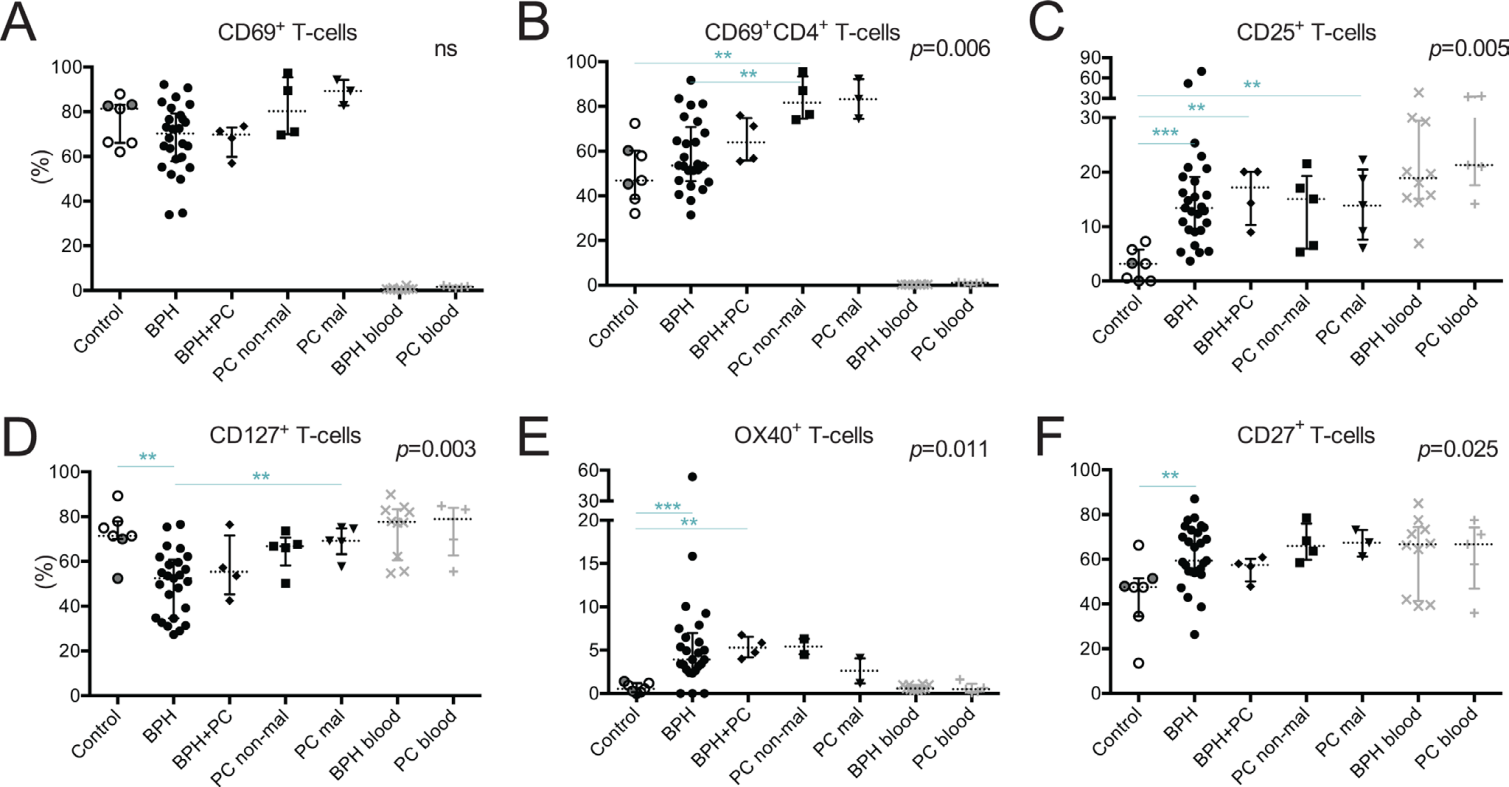
Characterization of T-cells in five different prostate conditions and peripheral blood Percentage of (**A**) T-cells expressing CD69; (**B**) CD4^+^ T-cells expressing CD69; (**C**) T-cells expressing CD25; (**D**) CD127; (**F**) OX40 (CD134); and (**E**) CD27 together with median and interquartile ranges. *P*-values for Kruskal-Wallis test on five prostate conditions are presented, peripheral blood values (BPH/PC blood) were excluded from all statistical analysis. Horizontal lines present the significant results of post-hoc Mann-Whitney *U*-tests. Due to multiple comparisons, Bonferroni correction was calculated and significance level of *p* < 0.01 was used. Values resulting in * (*p <* 0.05) were excluded, not plotted and considered as trends. **p < 0.01; ***p < 0.001, ns = no significance. Among the control prostates, the results of the two youngest donors are marked in dark grey circles.

CD69, generally considered as an early activation marker or as a marker for tissue-resident memory T-cells, was expressed by a majority of prostate-infiltrating T-cells compared to peripheral blood (Figure 3A). There was an increase in the percentage of CD4^+^ T-cells expressing CD69 in non-malignant tissue of PC patients compared to control tissue and BPH, where a large spread was observed (median 81.7% vs. 46.9% and 53.5% respectively, *p* = 006, *p* = 0.008) (Figure 3B).

### Cytokine and co-stimulatory receptors on T-cells

T-cells expressing CD25, the α-chain of the IL-2 receptor, were more abundant in malignant tissue of PC patients compared to control tissue (13.7% vs. 3.3%, *p* = 0.005) (Figure 3C). This was also a trend in the non-malignant tissues of PC patients (median 15.1% vs. 3.3%, *p* = 0.018). In accordance with these findings, BPH and BPH+PC samples also had significantly increased percentages of CD25^+^ T-cells compared to control prostates (median 13.4% and 17.2% respectively, *p* < 0.001 and *p* = 0.006) (Figure 3C).

Next, we looked at the proportion of T-cells expressing the α-chain of the IL-7 receptor, CD127. The proportion of CD127^+^ T-cells was decreased in BPH tissue compared to malignant PC lesions (median 52.5% vs. 69.2%, *p* = 0.007) and control prostates (median 52.5% vs. 71.4%, *p* = 0.001) (Figure 3D).

Two members of the TNFR family of co-stimulatory receptors, OX40 (CD134) and CD27, were present in higher frequencies of T-cells in BPH compared to control tissues (*p* < 0.001 and *p* = 0.006 respectively) (Figure 3E, 3F). The increase in OX40^+^ T-cells was also significant for the BPH+PC samples compared to controls (*p* = 0.006) (Figure 3E).

### Co-inhibitory receptor expression on T-cells

**Figure 4:**
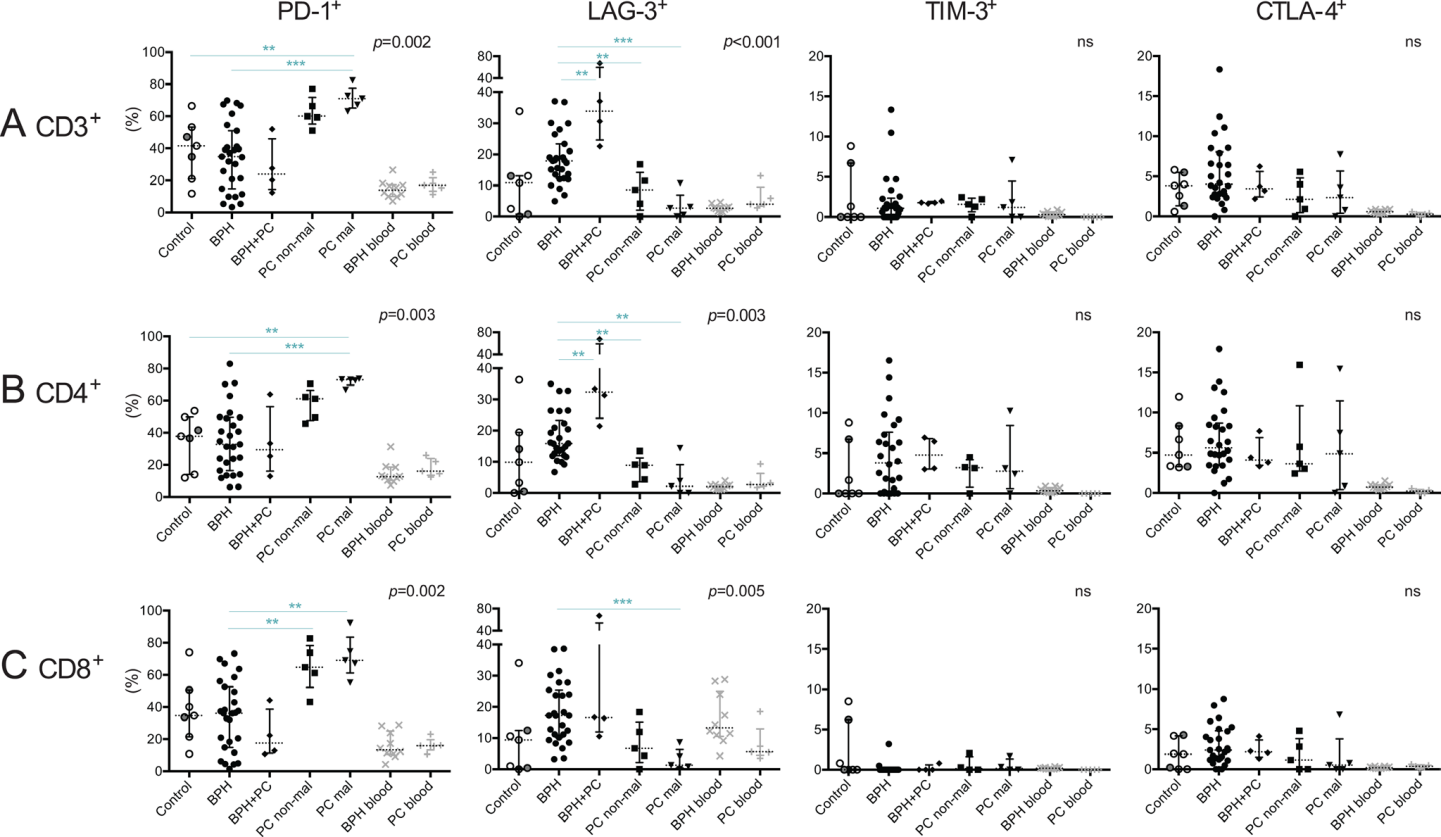
Characterization of T-cells expressing co-inhibitory receptors in five different prostate conditions and peripheral blood Percentage of PD-1^+^, LAG-3^+^, TIM-3^+^ and CTLA-4^+^ on (**A**) T-cells (CD3^+^); (**B**) CD4^+^ T-cells; and (**C**) CD8^+^ T-cells together with median and interquartile ranges. *P*-values for Kruskal-Wallis test on five prostate conditions are presented, peripheral blood values (BPH/PC blood) were excluded from all statistical analysis. Horizontal lines present the significant results of Mann-Whitney *U*-tests. Due to multiple comparisons, Bonferroni correction was calculated and significance level of *p* < 0.01 was used. Values resulting in * (*p <* 0.05) were excluded, not plotted and considered as trends. **p < 0.01; ***p < 0.001, ns = no significance. Among the control prostates, the results of the two youngest donors are marked in dark grey circles.

Expression of co-inhibitory receptors PD-1, LAG-3, TIM-3 and CTLA-4 was analyzed to obtain insight into the activation and functional status of prostate-infiltrating T-cells (Figure 4). In general, these receptors are up-regulated in response to activation and can be expressed continuously on T-cells in both chronic viral infections and tumors [21–24]. Upon binding with its cognate ligands, they can inhibit T-cell effector functions and proliferation. PD-1 was expressed by the highest percentage of T-cells in malignant PC lesions and was significantly different compared to control prostates (median 71.0% vs. 41.5%, *p* = 0.005) and BPH tissue (median 71.0% vs. 34.6%, *p* < 0.001) (Figure 4A). Both CD4^+^ and CD8^+^ subsets of T-cells were more frequent in malignant PC compared to BPH (PD1^+^CD4^+^ T-cells: median 73.0% vs. 32.7%, *p* < 0.001) (Figure 4B) (PD1^+^CD8^+^ T-cells: median 69.2% vs. 36.1%, *p* = 0.001) (Figure 4C). PD-1 was expressed by a similar proportion of total T-cells in the non-malignant PC lesions as observed in the malignant lesions (median 60.0% vs. 71.0%). The only significant difference between non-malignant lesions and other prostate conditions was comparing the proportion of PD1^+^CD8^+^ T-cells, which was decreased in BPH (median 64.8% vs. 36.1% *p* = 0.004) (Figure 4A–4C). For representative plots for all prostate conditions, see Supplementary Figure 1B.

Interestingly, the opposite was found for LAG-3, which was expressed by a larger proportion of T-cells in BPH (median 17.9%) and BPH with concurrent PC (median 33.8%) than in PC lesions (median 8.6% and 2.6% in non-malignant and malignant lesions respectively). Median percentage in control prostates was 10.9% (Figure 4A). This was also observed when analyzing CD4^+^ and CD8^+^ T-cell subsets separately (Figure 4B, 4C).

There were no statistical differences between the prostate conditions regarding percentage of T-cells or CD4^+^/CD8^+^ subsets of T-cells expressing TIM-3 or CTLA-4, which were found to be generally scarce (Figure 4A–4C).

### Lymphokine profile

**Figure 5:**
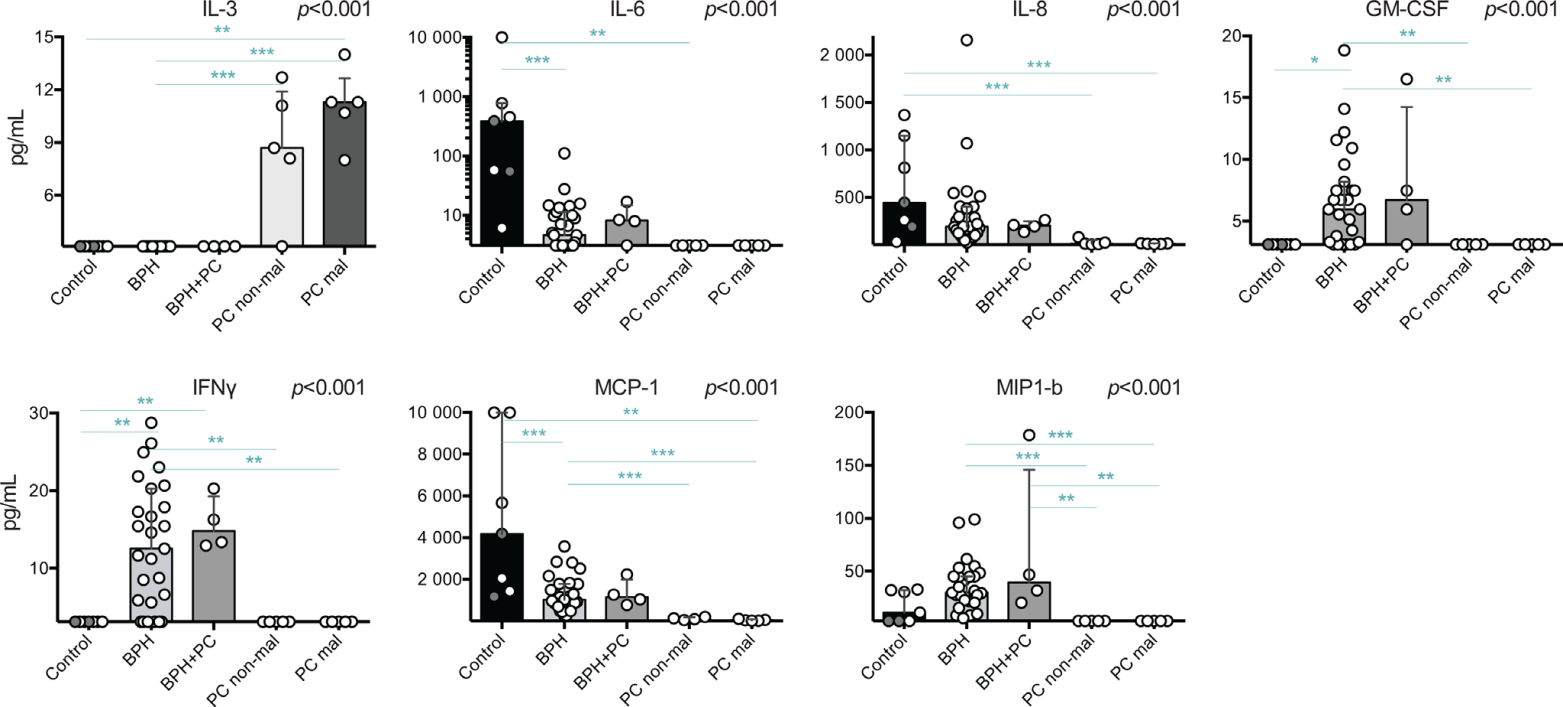
Significant findings of cytokine and chemokine profiling of prostate tissue supernatants by multiplex immunoassay Cytokines and chemokines with significant *p*-values from Kruskal-Wallis tests are presented. Individual samples are shown as circles and the bars represent the median value while the vertical line represents the interquartile ranges. Horizontal lines presents the results of the post-hoc Mann-Whitney *U*-tests. Due to multiple comparisons, Bonferroni correction was calculated and significance level of *p* < 0.01 was used. Values resulting in * (*p* < 0.05) were excluded, not plotted and considered as trends. **p < 0.01; ***p < 0.001. Among the control prostates, the results of the two youngest donors are marked in dark grey circles. Axis of IL-6 concentration is provided in a logarithmic scale

Supernatants of the processed tissue were analyzed by multiplex immunoassay. Out of 26 analyzed cytokines and chemokines, eight were significantly different in Kruskal Wallis tests: IL-3, IL-6, IL-8, GM-CSF, IFNγ, MCP-1 and MIP1-b (Figure 5). These were further evaluated by post-hoc analysis to elucidate between which conditions significant changes could be found.

IL-3 was only detected in PC tissue samples (both sites) and was significantly increased compared to BPH; median 11.3 and 8.7 pg/mL compared to below level of detection (< 3.2 pg/mL) in BPH (*p* < 0.001 for both). The level detected in malignant tissue was also significantly higher compared to control tissue which, similar to BPH, showed no detectable IL-3 levels (*p* = 0.008) (Figure 5). All values obtained below detection limit were adjusted to 3.1 pg/mL for statistical analysis and most likely reflects an overestimation of the actual concentration. In contrast, control tissue had the highest median concentrations of IL-6, IL-8 and MCP-1, which all, except IL-8, were undetectable in PC samples. BPH and BPH+PC samples showed similar levels of all selected lymphokines and had the highest concentrations of GM-CSF, IFNγ and MIP-1b compared to the other conditions (Figure 5). These were all undetectable in PC samples.

## DISCUSSION

In the current study, we isolated, quantified and characterized prostate-infiltrating immune cells by multicolor flow cytometry and assessed their tissue microenvironment by analyzing expression of cytokines using multiplex immunoassay. We did this for five different conditions of the prostate, aiming to elucidate the composition and phenotype of tissue-infiltrating T-cells and T-cell subsets, including their expression of co-inhibitory receptors.

Patients with several different cancer types have shown encouraging responses in clinical trials investigating the efficacy of checkpoint-inhibitors targeting CTLA-4 and/or PD-1. However, PC patients have not yet shown durable responses, except for one recently published small phase II study [17]. It is important to understand which cancer patients are likely to benefit and which patients are not suitable candidates for this type of therapy. As checkpoint inhibitors target the co-inhibitory receptors and aim to release their suppressive effects, thereby unleashing the anti-tumor effector functions, it is important to characterize patterns of co-inhibitory receptor expression in different cancer types. Here, we characterized and compared the phenotypes of tissue-infiltrating lymphocytes in malignant and non-malignant sites of the same prostate affected by adenocarcinoma.

Remarkably, the tissue microenvironment as well as the phenotype and composition of infiltrating T-cells were virtually identical in malignant and non-malignant sites from the same prostate. These findings can be interpreted in several ways. First, it is possible that the immuno-modulatory effects of a progressing tumor extend far beyond the visible physical limits of the malignant site and affect the entire organ. Second, T-cells found in PC-affected prostates may acquire their phenotype at the malignant site(s) and then populate the rest of the organ carrying the phenotypic imprint of their recent interactions with tumor cells and/or tumor specific microenvironment. Finally, changes in T-cell phenotype, which foster immune escape and tumor progression, such as upregulation of PD-1, may reflect other systemic processes in the organ not directly linked to tumor growth, such as induction of peripheral tolerance to prostate-specific antigens. Although it is difficult at this point to differentiate between these scenarios, it is clear that regulation of exhaustion markers on prostate resident T-cells is complex and might be unique for the organ.

Unexpectedly, two of the evaluated co-inhibitory receptors (PD-1 and LAG-3) were expressed by high proportions of T-cells in control prostates while the majority of these samples lacked expression of TIM-3. LAG-3 was expressed by a considerable proportion of T-cells isolated from BPH and BPH+PC samples where increased TIM-3 expression was also observed, consistent with chronic activation of T-cells in the inflammatory BPH environment. However, T-cells isolated from PC sites (both malignant and non-malignant) contained rather low percentages of LAG-3 or TIM-3-expressing cells in spite of very high content of PD-1^+^ T-cells. These data are consistent with the notion that T-cell infiltrates present in control, BPH or PC-affected prostates persist in very different microenvironments.

Several previous studies have established a link between the number of co-expressed inhibitory receptors and progressive loss of responsiveness, i.e. more profound exhaustion, in TILs or virus-specific T-cells during the course of tumor progression or virus persistence [21, 22, 25, 26]. Notably, the majority of TILs isolated from PC-affected prostates in our patient cohort expressed PD-1 but did not co-express multiple co-inhibitory receptors (LAG-3, TIM-3 and/or CTLA-4). However, current clinical experience indicates that PC-associated TILs respond to PD-1 blockage poorly, if at all. Consistent with suppressed functionality of PC-derived TILs, we failed to detect a majority of analyzed lymphokines from PC-derived samples with the exception of IL-3. Recent epigenetic studies demonstrated that the capacity of PD-1 blockade to promote transcriptional rewiring and revive functionality of exhausted T-cells is very limited under conditions of prolonged antigenic exposure and long-lasting PD-1 expression [27, 28]. In light of these findings, it is reasonable to propose that PD-1 expression characteristic even for lymphocytes present in healthy/benign prostate tissue contributes to the development of profound exhaustion in PC-derived TILs without involvement of additional co-inhibitory receptors.

Contradicting our results, Ebelt *et al*. has reported PD-1^+^ T-cells to be rare in healthy prostates [18]. In our results, the two youngest prostate donors (age 22 and 36) with normal prostate histology had a high proportion of PD-1^+^ T-cells (47.2% and 34.9% respectively), indicating that the results found in the control prostate group are not only due to presence of identified benign changes. It is clear that the role and function of PD-1 in the healthy prostate environment remains to be further elucidated using multiple techniques in future studies. Another surprising finding was that the presence of Tregs did not seem to be unique to the PC setting, but was found frequently in the prostate compared to peripheral blood regardless of prostate condition, also indicating a role in prostate homeostasis.

The largest infiltration in the prostate was by T-cells. The B-cell infiltration showed trends in a similar pattern as observed with T-cells, but to a much lower degree. The NK-cell infiltration was, similar to the B-cells, also limited compared to the T-cell infiltration but showed even less variability between the different conditions. Although there was a trend towards increased NK-cell numbers in malignant sites compared to control prostates, it appears that NK-cell migration to and/or persistence in prostate tissue were not significantly affected by various pathologies affecting this organ. Due to differences in anatomical sites of the prostate groups, one cannot rule out that differences observed were affected by different composition of processed tissue.

*Ex vivo* isolation and flow cytometric analysis of tissue-resident lymphocytes complements quantification by immunohistochemical scorings and counts per mm^2^ by providing an easily relatable measurement of cells per gram. Our results suggest that while T-cell infiltrates can be analyzed even from small amounts of prostate tissue samples, characterization of B- and NK-cell populations will require more sensitive techniques. It should be noted that the number of samples in which NK-cell numbers could be assessed in malignant tissue was restricted to only three samples. Yet, these three samples showed similar results and they provide insight into the feasibility of fresh tissue NK-cell isolation.

Further analysis of prostate tissue microenvironment revealed numerous differences in concentrations of lymphokines between the conditions of the organ. Interestingly, IL-3 was only detected in PC samples (both malignant and non-malignant sites) and could speculatively be used as an indicator of ongoing malignant transformation within the prostate upon prostate tissue biopsy. Validation of this finding in a larger cohort of patients could lead to development of a new biomarker of ongoing malignant transformation in the prostate which would be less dependent on the precision of biopsy. In general, there were few lymphokines detected in the PC samples. This could be due to low amount of collected tissue and/or be the result of decreased cytokine production by dysfunctional T-cells. Control prostates contained the highest median concentrations of IL-6, IL-8 and MCP-1, and BPH had the highest concentrations of GM-CSF, IFNγ and MIP-1b. It is difficult to speculate on the induction and progression of pathological conditions based on these findings. Many of these cytokines are associated with inflammation and have been shown to be produced by stromal and epithelial cells originated from BPH tissue causing both autocrine and paracrine effects [29–31]. In our previously published findings based on BPH material, IL-8 and MCP-1 were extensively discussed due to their association to clinical parameters (prostate size and serum prostate specific antigen levels) [20]. Intriguingly in the current study, the levels of these two lymphokines, and also IL-6, were found to be even higher in the control prostate tissues. It is interesting that the control prostates seem to have a microenvironment rich in inflammatory and chemoattracting components, however, it should be noted that due to the high age of some of the donors, this could be reflecting an undetected, but still ongoing BPH progression. However, the two youngest donors, which showed normal histology, also had high concentrations of these cytokines. Of interest is also the finding that IFNγ could only be detected in BPH samples. This might reflect a more dysfunctional state of infiltrating B- and T-cells in the PC setting as well as reduced activation in control tissue, which is as also supported by decreased percentages of T-cells expressing several activation markers.

The major limitation of the current study is the limited number of samples. It reflects the logistical difficulties associated with obtaining this kind of material but also reflects the value of results generated by our analysis. Furthermore, we have tried to minimize the extent of contamination of malignant tissue by adjacent non-malignant tissue as much as possible during the sample isolation procedure. The limited sample size influences the statistical power of the findings which, nevertheless, clearly warrant further larger studies evaluating the composition and expression of immune subsets in prostates of PC patients and healthy controls. The observed spread highlights the biological variation between patients and such spread would most likely be observed with a larger sample size, however this remains to be determined. We warrant future larger studies with particular focus on co-inhibitory and co-stimulatory receptor expression by infiltrating T-cells that should ideally include analysis of their functional capacity *ex vivo*. This will help to guide identification of suitable targets for future immunotherapy of PC.

In conclusion, we suggest that PD-1 may play a role in controlling the homeostasis of prostate tissue rather than in contributing to PC-associated immune-suppression. These findings may explain the limited efficacy of PD-1 blockade in PC and highlight the need for additional modes of immune intervention for efficient immunotherapy of the disease.

## MATERIALS AND METHODS

### Ethical statement

This study was approved by the Regional Ethical Review Board in Stockholm, Sweden (2010/158-31/2, 2013/2122-32, 2014/1912-32). All patients were informed about the study and gave their consent to participate and donate blood and/or prostate tissue upon time of surgery. Consent for organ donation for research purposes had been given by the deceased organ donor before time of death or consent was obtained from the donor's relatives after declaration of death.

### Prostate cancer samples

Five patients undergoing radical prostatectomy for prostatic adenocarcinoma (median age 65, Table 1) were enrolled at the Urology Clinic, Karolinska University Hospital Solna (Stockholm, Sweden). All patients were clinically staged as T2 and later on, pathologic staging ranged from pT2 (*n* = 3) to pT3a (*n* = 2) according to the TNM classification system (Table 1). Heparinized peripheral blood samples (18 mL) were collected at the day of enrollment, median 15 days (14–36 days) before surgery. On the day of the procedure, paired prostate samples were collected immediately upon arrival at the clinical pathology laboratory at Karolinska University Hospital Solna. A pathologist (L.E.) performed macroscopic examination, divided the prostate by a horizontal section and harvested malignant and non-malignant prostate tissue blocks by shaving with a scalpel blade from cut surfaces (median 0.03 g and 0.15 g respectively, Table 1). The sample location was noted on an anatomical map and the histology at the locations was later evaluated by microscopic examination. The samples were put in PBS (0.01M), transported to the laboratory and processing began within a total time of two hours after surgery.

### Control prostate samples

Control prostate material was obtained from seven deceased organ donors (median age 72, Table 2) in collaboration with the Department of Transplantation Surgery, Karolinska University Hospital Huddinge (Stockholm, Sweden). The prostates were surgically removed from donors and transported to the laboratory. Median time from cold perfusion of the donor until processing was 4.5 hours (range 3–6.5 hours). Importantly, the prostates were excluded from cold perfusion by ligation of common iliac arteries and veins. Median weight of collected prostate tissue was 54.8g (Table 2). Five to eight tissue pieces were resected from each prostate, fixed in 4% buffered formaldehyde, and sent to the clinical pathology laboratory for routine embedding and sectioning. The slides were stained with hematoxylin and eosin for later examination by a pathologist (L.E.). All specimens were histologically confirmed not to have any malignant transformation. Due to the high age of some of the donors, histology revealed some benign changes such as glandular atrophy, BPH and mild to moderate chronic inflammation (Table 2) but as these donors had not required medical care for their prostates, this material was considered to be adequate control material for the current study. The results of the two youngest donors (age 22 and 36) with normal histology are highlighted with a different color in Figures 1–5.

**Table 2:** Control prostate donor characteristics and sample collection information

Donor #	Age	Cause of death	Processed prostate tissue (g)	Pathology comment
1	72	Cerebral hemorrhage	54.8	BPH
2	83	External trauma	59.5	BPH, mild chronic inflammation
3	77	Cerebral hemorrhage	60.0	Mild chronic inflammation, minor area of HG-PIN
4	22	External trauma	9.5	Normal histology
5	81	Cerebral hemorrhage	55.2	BPH, moderate chronic inflammation
6	53	Cerebral hemorrhage	48.1	Atrophy
7	36	Cardiac arrest	29.3	Normal histology

**Abbreviations:** BPH, benign prostatic hyperplasia; HG-PIN, high-grade prostatic intraepithelial neoplasia.

### Benign prostatic hyperplasia samples

BPH prostate samples from 31 patients (median age 72, Table 3) undergoing surgery by transurethral resection of the prostate (TURP) (*n* = 25) or transvesical enucleation (*n* = 6) were collected, processed and analyzed as previously published [20]. More extensive patient characteristics can be found in Norström *et al* [20]. The data from these patients is included in the present study as a benign reference material to compare different pathological conditions of the prostate. Four of the patients were diagnosed with concurrent PC (total Gleason score 6) in the pathological anatomical diagnosis of the material sent from their surgical procedures (Table 3). These patients are treated as a separate group in this study and abbreviated BPH+PC.

**Table 3:** Benign prostatic hyperplasia (BPH) patient characteristics and sample collection information

Patient #	Clinical factors				Pathology	Sample collection
	Age	Pre s-PSA (μg/L)	Prostate size (g)	Resected weight (g)	PAD	Collected weight (g)
1	83	127	N/A	4.3	BPH	2.3
2	66	11	85	34	BPH	5
3	81	5	45	5	BPH	2.3
4	92	0.6	32	10	BPH	2
5	64	3.8	62	20	BPH	5.2
6	68	3.3	37	20	BPH	10
7	75	3.4	42	15	BPH	5
8	56	20	70	25	BPH	5.1
9	68	9	65	30	BPH	10
10	93	N/A	57	22	BPH, PC	10
11	77	1	33	27	BPH	6
12	62	N/A	54	32	BPH	10
13	66	7.1	61	35	BPH, PC	10
14	66	11	62	35	BPH	10
15	83	N/A	37	7	BPH	3
16	77	N/A	40	15	BPH	7
17	75	0.8	35	30	BPH	10
18	75	11	65	48	BPH, PC	10
19	74	5	37	26	BPH, PC	10
20	74	5.6	N/A	23	BPH	5.2
21	72	N/A	30	20	BPH	8.2
22	66	N/A	N/A	8	BPH	3
23	62	4.4	48	25	BPH	10
24	63	4	66	32	BPH	10
25	77	6	83	36	BPH	10
26*	71	8.7	115	87	BPH	15
27*	66	150	120	84	BPH	27
28*	79	N/A	180	92	BPH	21
29*	69	N/A	N/A	144	BPH	13
30*	78	4	107	98	BPH	7.5
31*	63	3.8	130	N/A	BPH	3.5

**Abbreviations:** *resection performed with transvesical enucleation, all else were performed with transurethral resection of the prostate; s-PSA, serum prostate-specific antigen; N/A, not available; PC, prostate cancer with Gleason score 3 + 3; PAD, pathological anatomical diagnosis. Prostate size was determined as estimated weight evaluated by transrectal ultrasound prior to surgery.

### Processing, acquisition and analysis of prostate tissues and peripheral blood

Processing of the prostate tissues obtained from PC patients and deceased donors began immediately upon arrival to the lab and was performed as previously described [32]. Briefly, the processing combined mechanical dissociation and density gradient centrifugation for the isolation of prostate-infiltrating lymphocytes. No enzymatic digestion was used for the isolation of immune cells. Human BD Fc Block (BD Biosciences) was added according to manufacturer's instructions. Next, cells were added to a 96-well plate to which antibodies of the nine-color characterization panel had already been added (Supplementary Table 1). In some cases the panel was reduced due to low cell numbers. After 20 min of staining at 4^°^C, the cells were washed once with PBS and viability dye 7-AAD (BD Biosciences) was added according to manufacturer's instructions. The samples were acquired on a BD Canto I SORP with BD FACSDiva Software v.7.0 (BD Biosciences). Data was analyzed using FlowJo v.10.2 (Tree Star Inc.). FMO isotype controls were used for gating and background was removed. Gates for singlets, live cells, lymphocytes and CD3^+^ T-cells were used to characterize expression of indicated surface markers (Supplementary Figure 1A). Also, where indicated, subsets of CD4^+^ and CD8^+^ T-cells (CD3^+^/CD4^+^or CD8^+^/) were analyzed for surface markers.

Peripheral blood samples were processed at the day of collection or the following day, in the latter case after being stored at room temperature in the dark overnight. The blood was diluted twice in PBS and put on a density gradient using Lymphoprep (1.077g/cm^2^, Fresenius Kabi) at a ratio of 1:3 for 20 min at 800 *g*. The cells were stained, acquired, and analyzed by flow cytometry as described above.

### Lymphokine profiling of supernatants

Fluorescent bead-based multiplex immunoassay was performed on supernatants collected during processing of the prostate tissues using MILLIPLEX MAP Human Cytokine/Chemokine Premixed 26 Plex (Millipore Corporation) as previously published [20, 33, 34]. All samples were processed in the same volume of PBS (50 mL) from which supernatants were used for the multiplex assay. The values obtained above or below the range of the standard curves (3.2–10 000 pg/mL) were adjusted to 3.1 and 10 001 pg/mL respectively as the precise concentrations of these samples cannot be known.

### Statistical analysis

Peripheral blood samples were excluded from all statistical analysis and were used as a reference material in Figures 2, 3, 4. Phenotypic and cytokine profiles were initially compared between the five different prostate conditions using non-parametric unpaired Kruskal Wallis test. The significant findings (*p* < 0.05) were further analyzed using non-parametric unpaired two-tailed Mann-Whitney *U*-test comparing two conditions at a time. When comparing malignant and non-malignant PC samples, non-parametric paired two-tailed Wilcoxon signed rank test was also used but did not result in any significances due to low sample numbers. Due to multiple comparisons post hoc, the significance level was adjusted to *p* < 0.01 as calculated with Bonferroni correction (0.05/5 groups). All values resulting in * (*p <* 0.05) were therefore considered as trends and were not plotted with the exception of Figure 1. The differences were considered statistically significant if *p* < 0.01 (**) or *p* < 0.001 (***). All percentages, counts and concentrations are presented as median values. Prism 6.0 (GraphPad Software Inc.) and Microsoft Excel 14.7 (Microsoft Corporation) were used for all statistical analysis.

### Quantification of lymphocyte subsets

T-cell counts were obtained by pooling the total number of T-cells (CD3^+^ events) obtained from each prostate tissue sample by flow cytometry and divided by the collected sample weight. This resulted in a value reflecting number of T-cells per gram prostate tissue. The counts of B- and NK-cells were calculated in a similar way as for T-cells but from the acquired CD3^−^ cells and number of CD19^+^ and CD56^+^ events respectively. All mentioned values of median cells per gram have been rounded to the nearest 10.

## SUPPLEMENTARY FIGURE AND TABLE



## Abbreviations

BPH: benign prostatic hyperplasia
BPH+PC: BPH with concurrent PC
CTLA-4: cytotoxic T-cell antigen 4
LAG-3: lymphocyte activation gene-3
mal: malignant
NK: natural killer
non-mal: non-malignant site of prostate affected by PC
PD-1: programmed cell death 1
PD-L1: PD-1 ligand 1
TIM-3: T-cell immunoglobulin and mucin-domain containing-3
Treg: regulatory T-cells
TURP: transurethral resection of the prostate
PC: prostate cancer
TILs: tumor-infiltrating lymphocytes
