# Supplementary material for: Characterization of infiltrating lymphocytes in human benign and malignant prostate tissue
Source: Oncotarget. 2017 Jul 24;8(36):60257–69. doi: 10.18632/oncotarget.19528 (PMC5601136; doi:10.18632/oncotarget.19528)
Supplement: Supplementary file 1 [file oncotarget-08-60257-s001.pdf]

Characterization of infiltrating lymphocytes in human benign and malignant prostate tissue

SUPPLEMENTARY MATERIALS

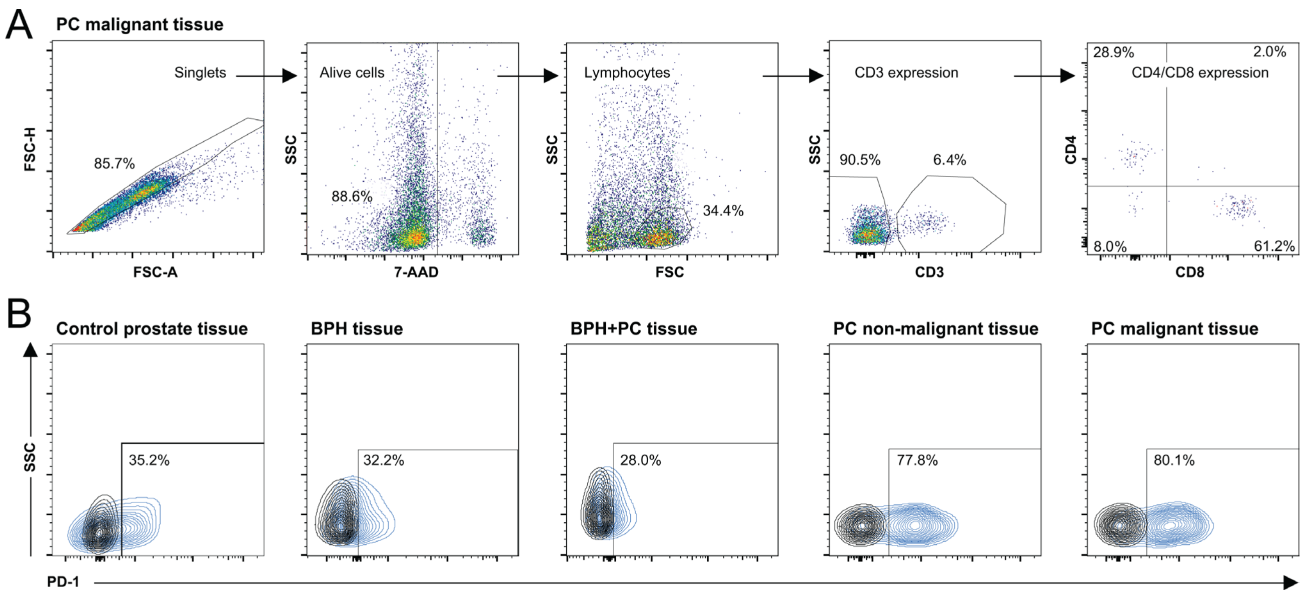

Supplementary Figure 1: Example plots from flow cytometric analysis. (**A**) General gating strategy used for all samples: singlets, living cells, lymphocytes, $CD3^+$ T-cells and then further markers, here CD4 and CD8 are shown. Plots are from prostate cancer (PC) patient 4 (malignant tissue). (**B**) Representative plots of PD-1^+ T-cells (gated from $CD3^+$ T-cells) from five prostate conditions. Isotype controls from respective prostate condition are shown in black and PD-1 expression is shown in blue from indicated prostate condition

Supplementary Table 1: Complete list of all antibodies used for extracellular characterization of cells

Company	Target	Clone	Fluorochrome
BD Biosciences	CD28	CD28.1	FITC
	CD69	FN50	FITC
	CD94	HP-3D9	FITC
	CD134/OX40	ACT35	FITC
	CD158b	CH-L	FITC
	CD279/PD-1	MIH4	FITC
	Isotype IgG1	X40	FITC
	CD25	M-A251	PE
	CD27	M-T271	PE
	CD56	NCAM16.2	PE
	CD279/PD-1	MIH4	PE
	Isotype IgG1	X40	PE
	CD197/ CCR7	150503	PE-CF594
	Dead cells	-	7-AAD
	CD3	SK7	PE-Cy7
	CD19	HIB19	APC
	CD45RO	UCHL1	APC
	Isotype IgG1	X40	APC
	CD8	SK1	APC-Cy7
	CD4	RPA-T4	V500
Beckman Coulter	TCRγδ pan	IMMU510	FITC
	CD127	R34.34	APC-A700
BioLegend	Isotype IgG2a	MOPC-173	FITC
LifeSpan Biosciences	CD137/4-1BB	4B4-1	FITC
	CTLA-4	A3.4H2.H12	FITC
	CD223/LAG-3	17B4	FITC
	CD366/TIM-3	F38-2E2	APC
Miltenyi Biotec	CD137/4-1BB	4B4-1	FITC
	CD159/NKG2A	REA110	FITC
	CD223/LAG-3	REA351	PE
	CD366/TIM-3	F38-2E2	APC
